# Therapeutic Effects of Bee Bread on Obesity-Induced Testicular-Derived Oxidative Stress, Inflammation, and Apoptosis in High-Fat Diet Obese Rat Model

**DOI:** 10.3390/antiox11020255

**Published:** 2022-01-28

**Authors:** Joseph Bagi Suleiman, Mahaneem Mohamed, Ainul Bahiyah Abu Bakar, Zaida Zakaria, Zaidatul Akmal Othman, Victor Udo Nna

**Affiliations:** 1 Department of Physiology, School of Medical Sciences, Universiti Sains Malaysia, Kubang Kerian 16150, Kelantan, Malaysia; bagisuleiman@student.usm.my (J.B.S.); ainul@usm.my (A.B.A.B.); zaida_zakaria@student.usm.my (Z.Z.); zaidaakmal@unisza.edu.my (Z.A.O.); 2 Department of Science Laboratory Technology, Akanu Ibiam Federal Polytechnic, Unwana P.O. Box 1007, Ebonyi State, Nigeria; 3 Unit of Integrative Medicine, School of Medical Sciences, Universiti Sains Malaysia, Kubang Kerian 16150, Kelantan, Malaysia; 4 Unit of Physiology, Faculty of Medicine, Universiti Sultan Zainal Abidin, Kuala Terengganu 20400, Terengganu, Malaysia; 5 Department of Physiology, Faculty of Basic Medical Sciences, College of Medical Sciences, University of Calabar, Calabar P.O. Box 1115, Cross River State, Nigeria; victorudon@unical.edu.ng

**Keywords:** oxidative stress, inflammation, apoptosis, bee bread, testis

## Abstract

Obesity is a debilitating disorder with a variety of problems including oxidative stress, inflammation, and apoptosis. The aim of our study was to investigate the therapeutic role of bee bread on oxidative stress, apoptosis, and inflammation in the testis of obese rats. Thirty-two adult male *Sprague Dawley* rats, with weights between 230–300 g, were distributed into four groups (n = 8/group), namely normal control (C), obese (Ob), obese + BB or obese + OR [high-fat diet (HFD) for 6 weeks then HFD plus bee bread or orlistat for another 6 weeks] groups. Bee bread (0.5 g/kg) or orlistat (10 mg/kg/day) was diluted with distilled water and administered daily for 6 weeks by oral gavage. There were significant decreases in the activities of antioxidant enzymes [glutathione-S-transferase (GST), superoxide dismutase (SOD), glutathione peroxidase (GPx), catalase (CAT), glutathione reductase (GR)], glutathione (GSH)] and total antioxidant capacity (TAC) levels and mRNA expressions of nuclear factor erythroid 2–related factor 2 (*Nrf2*), superoxide dismutase (*Sod*), catalase (*Cat*) and glutathione peroxidase *(Gpx*) in the obese group relative to the control group. Meanwhile, the mRNA levels of pro-inflammatory markers, namely: inducible nitric oxide synthase (*Inos*), nuclear factor kappa B (*Nf-κβ*), tumour necrotic factor α (*Tnf-α*) and interleukin 1β (*Il-1β*) were significantly increased while interleukin (*Il-10*) was decreased in the obese group relative to the control group. Further, proliferating cell nuclear antigen (PCNA) immunoexpressions decreased while cleaved caspase-3 immunohistochemical staining increased significantly in the obese group, in addition to increases in the mRNA levels of *p53*, *Bax*, *Caspases-8*, *9* and *3*, relative to the control group. Treatment with bee bread showed increases in antioxidant enzymes and PCNA immunoexpression, as well as decreases in inflammation and apoptosis markers in the testes. This study has shown that bee bread has therapeutic effects against oxidative stress, inflammation, apoptosis in the testis of HFD-induced obese male rats, thereby suggesting its role as a natural supplement capable of treating obesity-induced male reproductive impairment.

## 1. Introduction

Bee bread is usually formed due to the outcome of the fermentation from the combination of pollen, digestive enzymes found in saliva, and nectar [1,2]. It possesses high protein, carbohydrates, fats, and mineral components. Bee bread comprises of 24–35% carbohydrates, 20% protein, 3% lipids, 3% minerals and vitamins, enzymes (phosphatases, glucose-oxidase, saccharase and amylase), amino acids (proline, glutamic acid, aspartic acid, histidine, arginine, valine, isoleucine, leucine, methionine, tryptophan, lysine, threonine, cysteine, phenylalanine, alanine, glycine, tyrosine and serine), carotenoids, phenolic acid and flavonoids, pantothenic acid, polyphenols, and sterols [3]. The possession of large amounts of protein and amino acid is due to the fact that bee pollen also possesses a high amount of these nutrients. It has also been found that the amount of protein in bee bread depends largely on the type of flower, pollen grains and region where they are located [3]. The presence of phenolic acids (caffeic acid, gallic acid, trans 3-hydroxycinnamic acid, trans-ferulic acid, 2-hydroxycinnamic acid) and flavonoids (apigenin, kaempferol, quercetin, and mangiferin) have been found in our study [3].

Bee bread also has several health benefits including antioxidant [4,5], anti-inflammatory [6], anti-apoptotic [7], antimicrobial [3,8], anticancer [9], and immunological effects [10,11] on various tissues. Its effects on the male reproductive system cannot be over emphasised; our previous study has revealed that concurrent administration of a high-fat diet and 0.5 g/kg bee bread for twelve weeks showed protective effects against testicular oxidative stress, inflammation, and apoptosis in rats [12].

Obesity is defined as a combination of various factors (oxidative stress, inflammation, and apoptosis) giving rise to increased levels of fat in the body which results in a disease with detrimental consequences, such as hypertension, high LDL cholesterol, low HDL cholesterol, or dyslipidemia, Type 2 diabetes, coronary heart disease, stroke, gallbladder disease, Osteoarthritis, and even mortality [13]. The prevalence of obesity has reached a pandemic level in the last 50 years [14,15]. The serious detrimental effects of obesity have been seen on the brain [16], thyroid gland [17], heart [18], kidneys [19], liver [20], pancreas [21], spleen [22], skeletal muscles [23], bones [24], female reductive system [25] and the male reproductive system [26].

Oxidative stress evolves from the excessive production of the radical oxygen species (ROS) as a result of the imbalance between its production and elimination by the antioxidant system. The radical oxygen reactive species (ROS) includes a hydroxyl ion (OH^−^), hydrogen peroxide (H_2_O_2_) and a superoxide anion (O_2_^−^). During obesity, oxidative stress in the testis of males is heightened, as seen in both clinical [27,28] and experimental studies [29,30,31]. Likewise, inflammation plays a role in testicular dysfunction because of the imbalance between the pro-inflammatory and anti-inflammatory cytokines [32]. It has been implicated in the testis of most patients with metabolic diseases such as obesity [26]

Apoptosis as a process of programmed cell death has been studied in detail in the past, especially in the testis where about 75% of the germ cells are eliminated via the process. The process of apoptosis is irreversible with the activation of caspase, and a cell is committed to death while the engulfment genes remove the dead cell. It is important to note that the precise mechanisms of male germ cell apoptosis is yet to be established because of the web of interactions of different types of cells within the testis and their ability to respond to stimuli [33,34].

Therefore, the motive of this study was to examine the possible therapeutic effects of bee bread on oxidative stress, inflammation, and apoptosis in the testis of obese rats, whereby rats were fed with a high-fat diet (HFD) for 6 weeks to induce obesity and then treated with bee bread for another 6 weeks.

## 2. Materials and Methods

### 2.1. Animal Handling

Male *Sprague Dawley* rats, aged 10 weeks old, measuring between 230 and 300 g, all 32 in number, were procured from the Animal Research and Service Centre (ARASC), Universiti Sains Malaysia (USM), Kelantan, Malaysia. The rats were brought to the laboratory of the Department of Physiology, School of Medical Sciences, USM, Malaysia and kept in animal room with 12/12-h reversed light/dark cycle and photoperiods at 12:00 p.m. to 12:00 a.m., and the temperature was regulated at 23 ± 2 °C. Just before the start of the study, rats were acclimatised for 1 week. Meanwhile, animals were allowed free access to rat pellets or HFD and water. Ethical approval (USM/IACUC/2018/(113)(933) was issued by USM Animal Ethics Committee. Instructions on animal handling and care were strictly followed according to the National Institute of Health.

### 2.2. Materials

#### 2.2.1. Preparation of Bee Bread Samples

Fresh bee bread, obtained from *Heterotrigona itama* stingless bees, was bought from Mentari Technobee PLT (bee farm) in Kota Bharu, Kelantan, the East coast region of Malaysia, south-east Asia. It was gathered from the month of January to March, which corresponds to the dry season in Malaysia. However, the bee bread was taken to the Physiology Laboratory, USM, Malaysia, and the initial weight was documented. Thereafter, the final weight was also determined after samples were dehydrated with the aid of a food dehydrator at 35 °C for 4 h until no more change in weight was observed. They were then blended into powder form and stored at −20 °C until use.

#### 2.2.2. Diets

Standard rat pellets were bought from ARASC, USM, its constituents were 19.2% protein, 6.1% carbohydrate, 4.1% fat, 6.9% ash, 35.9% polysaccharides, 11.3% moisture, 4.9% disaccharides, trace elements, vitamins, minerals, amino acid, while the HFD was prepared following methods described by Othman et al. [35], which was made up of 300 mg calcium, 100 UI vitamin D3, 12% cholesterol, 32 g ghee, and 68 g of powered standard rat pellets.

#### 2.2.3. Chemicals and Kits

SensiFAST SYBR Hi-ROX One-Step PCR kit (Bioline, London, UK) (BIO-73005), as well as RNAlater, Acridine orange, and agarose (Sigma-Aldrich, St. Louis, MO, USA). Innu-Prep RNA mini kit Analytik (Jena, Germany), primers (Integrated DNA Technologies, IDT, Selangor, Malaysia), PCR tubes and strips (Applied Biosystems, Life Technologies, Dongguan, China). Proliferating cell nuclear antigen (PCNA) (PAA591Mi01), caspase-3 (PAA626Ra01), tumour necrosis factor-alpha (TNF-α) (PAA133Ra01), nuclear factor kappa B (NF-κB) (PAB824Ra01), IL-1β (PAA563Ra01), and IL-10 (PAA056Ra01) rabbit polyclonal primary antibodies, were obtained from Cloud-Clone (Cloud-Clone Corp, Katy, TX, USA), while Dako EnVisionTM System/HRP, Rb (DAB) for signal detection was obtained from Agilent Technologies, Inc. (Santa Clara, CA, United States of America). Orlistat was obtained from Catalent Australia Pty Ltd., Australia. Other chemicals like [Picric acid (Sigma, St. Louis, MO, USA), formaladehyde (Merck, Darmstadt, Germany), acetic acid (Leica biosystems, United States of America] used for Bouin’s solution, paraffin (Thermo Scientific, Bremen Germany), Haris hematoxylin solution (Sigma-Aldrich, St. Louis, MO, United States of America) and alcohol (Merck, Darmstadt, Germany) were of analytical grade.

### 2.3. Study Design

The rats (males) were fixed into four groups randomly (n = 8/group) as stated below:

- Normal Control (C) group: administered with normal rat pellets and distilled water (1 mL) once daily for 12 weeks.
- Ob group: administered with HFD and 1 mL distilled water once daily for 12 weeks.
- Ob + BB group: administered with HFD for 6 weeks and then HFD + bee bread (0.5 g/kg b.w./day) for another 6 weeks.
- Ob + OR group: administered with HFD for 6 weeks and then HFD + orlistat (10 mg/kg b.w./day) for another 6 weeks.

Bee bread and orlistat [120 mg of the active ingredient: orlistat, and the inactive ingredients: sodium starch glycolate, microcrystalline cellulose, talc, povidone, and sodium lauryl sulphate (standard drug)] doses were selected based on our previous studies [35,36].

### 2.4. Bodyweight, Absolute and Relative Organ Weights

Throughout the period of experiment, weights of rats were recorded every week and on the last day prior to sacrifice. Seminal vesicles, penis, prostate, testes, and epididymis (male reproductive organs) were detached, rinsed, and weighed. The relative weights were computed and presented as a proportion of the final bodyweight. Values for Lee obesity index were terminated at 315 while values for body mass index were terminated at 0.75 gcm^−2^ [37,38,39].

### 2.5. Determination of Anthropometrical and Nutritional Parameters

After the expiration of 12 weeks experiment, abdominal circumference (AC) and thoracic circumference (TC) and naso-anal length (NAL) were measured with rats placed under anesthesia. The energy intake (Energy intake = mean food consumption × dietary metabolisable energy), Lee obesity index ($\mathrm{Lee}\;\mathrm{obesity}\;\mathrm{index}=\frac{3\sqrt{\mathrm{body}\;\mathrm{weight}\;\left(g\right)\;}}{\mathrm{NAL}\;\left(\mathrm{cm}\right)}\times 1000$) and BMI ($\mathrm{BMI}=\frac{\mathrm{body}\;\mathrm{weight}\;\left(g\right)}{\;{\left(\mathrm{Length}\right)}^{2}{\left(\mathrm{cm}\right)}^{2}}$), were computed using values obtained from NAL body weights, and food consumption.

### 2.6. Sample Collection

During sacrifice, the left testis was divided into two parts. Ten percent homogenate was prepared using one part after being immersed in Tris-HCl buffer (pH 7.4) and then centrifuging at 4000× *g* for 15 min at 4 °C, whereas the other half was put in RNAlater and stored at −80 °C for future use. The right testis was also harvested for the histological study.

### 2.7. Determination of Serum Lipid Parameters

To determine lipid parameters, blood from the abdominal aorta was utilised. Briefly, a centrifuge tube containing blood was made to stand for 1 h, after which the separation of serum was carried out using a centrifuge for 10 min at 1400× *g*. Total cholesterol (TC) was evaluated by enzymatic method [Equipment: ARCHITECT ci8200 System], while high-density lipoprotein cholesterol (HDL-C) was evaluated by the elimination of chylomicron (CM) [Equipment—ARCHITECT ci8200 System]. Triglyceride by enzymatic methods of Glycerol Phosphate oxidase [Equipment—ARCHITECT c System and ARCHITECT c Triglyceride assay file] and direct calculation was employed to calculate LDL-C from above.

### 2.8. Histopathology of the Testis

After the sacrifice, Bouin’s solution was used to preserve the detached right testis for 24 h, meanwhile, different grades of alcohol (70, 95 and 100%) were used to dehydrate it and then placed in blocks of paraffin. The microtome was used to Section 5 µm thickness of tissue, while hematoxylin and eosin (H & E) technique was used for the staining process. Slides were observed under a light microscope (Olympus BX41, Olympus Corporation, Tokyo, Japan), and quantitative data obtained from images were analysed using Image Analyser software (Soft Imaging System, VGA utilities version 3.67e, Tokyo, Japan) at a magnification of ×100.

### 2.9. Oxidative Stress Status of the Testis

Oxidative stress status was determined by evaluating the aliquot of the left testis homogenate which was stored in −80 °C for the following parameters, glutathione-S-transferase (GST), glutathione peroxidase (GPx), malondialdehyde (MDA), total antioxidant capacity (TAC), superoxide dismutase (SOD), catalase (CAT), glutathione reductase (GR), and glutathione (GSH) level. Concisely, methods of Ohkawa et al. [40] were used to assess MDA levels, and methods of Koracevic et al. [41] were used to assess TAC. Meanwhile, the enzyme activities of SOD were evaluated by methods of Sun et al. [42], and CAT was evaluated by methods of Goth [43]. Further, enzyme activity of GPx was determined by methods as described by Paglia and Valentine [44], and GST was evaluated by methods of Habig et al. [45], respectively, while GR activity was evaluated by methods of Carlberg and Mannervik [46], and GSH activity was evaluated by methods of Annuk et al. [47]. Similarly, ELISA kit (Elabscience, United States of America, Catalog No: E-EL-R0520) was used to evaluate inducible nitric oxide synthase (iNOS) activity and the total protein assay kit (Elabscience, United States of America, Catalog No: E-EL-R0520) was used to evaluate total protein level.

### 2.10. Evaluation of Intratesticular Levels of Lactate, Lactate Dehydrogenase and Glucose

Following manufacturer’s instructions, the evaluation of lactate, lactate dehydrogenase and glucose levels were carried out with the aid of ELISA kits (Elabscience, USA), available commercially.

### 2.11. RNA Expression for Antioxidant, Inflammatory and Apoptosis Markers

#### 2.11.1. Extraction, Quality, and Purity of RNA

Based on the maker’s instructions, total RNA was evaluated using Innu Prep RNA mini kit (Analytik Jena, Jena, Germany). Similarly, twenty mg of testis tissue was used to determine the purity and concentration of samples with the aid of spectrophotometer (Eppendorf Nanodrop BioPhotometer plus, Stevenage, United Kingdom). Samples with OD of 260/280 and 1.8–2.0 were used to know samples without impurities. Meanwhile, samples that were pure were exposed to gel electrophoresis with application of 1% agarose (*w*/*v*) in 1× LB buffer and observed with the aid of a UV transilluminator (ChemiDoc XRS, Bio-Rad Laboratories, Hercules, CA, USA), and pure samples were employed during the RT-qPCR amplification.

#### 2.11.2. Real-Time RT-qPCR

RT-qPCR was evaluated using the StepOnePlus Real-Time PCR system (Applied Biosystems Co., Foster City, CA, USA) based on manufacturer’s protocol. The melt curve and standard curve were used to determine their specificity and efficiencies after Integrated DNA Technologies (IDT, Malaysia) combined primers were selected from the gene bank (Table 1). Further, the 3-step cycling was conducted using the PCR machine [SensiFAST SYBR Hi-Rox One-Step PCR kit (Bioline, United Kingdom)]. Briefly, initial denaturation finished at 95 °C for 2 min, then 40 cycles of denaturation for 5 s at 95 °C, followed by annealing for 10 s at 60 °C, and extension for 5 s at 72 °C. A housekeeping gene [Glyceraldehyde-3-phosphate dehydrogenase (GAPDH)] was utilised, while the 2^ΔDDCt^ method was used for relative quantification [48].

### 2.12. Immunohistochemistry Expression for PCNA, TNF-α, IL-1β, IL-10 and Cleaved Caspase-3

Slides having 4 μm thick testis sections attached to them were immersed in a pressure cooker filled with tris-EDTA buffer and 0.05% tween 20 (pH 9.0), to retrieve antigens for 3 min. Thereafter, endogenous peroxidase was blocked using 3% hydrogen peroxide in phosphate buffered saline for 5 min, followed by washing with distilled water and tris-buffered saline containing 0.05% tween 20 (TBST, pH 8.4). Further, slide containing testis sections were incubated with polyclonal primary antibodies for PCNA (1:50), cleaved caspase-3 (1:150), NF-κB (p65) (1:80), IL-10 (1:40), IL-1β (1:80), and TNF-α (1:120) (Cloud-Clone Corp, USA) for 24 h at 4 °C. Testis sections were incubated with Dako EnVision™+ System/HRP labelled polymer containing goat anti-rabbit secondary antibody (Agilent Technologies, Inc. United States of America) for 30 min at room temperature after washing with TBST. With the aid of Dako 3,3′-diaminobenzidine substrate (Agilent Technologies, Inc. USA) visualisation was conducted for 5 min at room temperature. Thereafter, hematoxylin was employed to counter stain the sections for 5 s. They were dehydrated and viewed using a light microscope (Olympus BX41, United Kingdom). ImageJ software (ImageJ, NIH—Bethesda, MD, USA) was used to analyse the photographs for area and intensity of brown stain, and also to evaluate the quantitative data of the stained area.

### 2.13. Statistical Analysis

All data were analysed with the aid of GraphPad Prism version 7.0 (GraphPad Software Inc., La Jolla, CA, USA). All values were assessed for normality and homogenous variance using Shapiro–Wilk and D’Agostino–Pearson Omnibus normality tests. One-way analysis of variance (ANOVA) was employed for values with normal distribution, followed by Tukey post-hoc test. Values are shown as mean ± standard deviation (SD). *p* < 0.05 was considered statistically significant.

## 3. Results

### 3.1. Body Weights and Weight Gain

The Ob group revealed significant increases in the final body weight and weight gain relative to the C group (*p* < 0.05). Meanwhile, the weight gain showed a significant decrease in the Ob + BB group, but not in the Ob + OR group relative to the Ob group (*p* > 0.05) (Table 2).

### 3.2. Energy Intake, Feed Consumption, Lee Obesity and Body Mass Index

BMI and the Lee obesity index significantly increased in the Ob group relative to the C group (*p* < 0.05). Bee bread and orlistat treatment in the Ob + BB and Ob + OR groups, respectively, showed significant decreases in the above parameters relative to the Ob group (Table 3). Further, energy intake was significantly increased in the Ob, Ob + BB and Ob + OR groups relative to the C group (*p* < 0.05) (Table 3).

### 3.3. Effects of Bee Bread on Weight of Reproductive Organs and Epididymal Fat of Obese Rats

In the Ob group, the absolute and relative weights of the testis decreased significantly relative to the C group (Table 4). Moreover, significant increases were observed in absolute and relative weights of epididymal fat in the Ob group relative to the C group. Treatment with bee bread also showed a significant decreased relative weight of epididymal fat in the Ob + BB group relative to the C and Ob groups. However, there were no significant differences among groups in the relative weights of the prostate, seminal vesicle, epididymis, and penis (Table 4).

### 3.4. Effects of Bee Bread on Serum Lipid Profile of Obese Rats

The levels of triglyceride, LDL-C, total cholesterol increased significantly in the Ob group relative to the C group (*p* < 0.05), and similarly, the Ob group revealed a significant decrease in HDL level relative to the C group (Table 5). The bee bread-treated group showed significant amelioration in HDL-C and LDL-C levels relative to the Ob group (*p* < 0.05) (Table 5).

### 3.5. Effects of Bee Bread on Testicular Histology of Obese Rats

**Figure 1:**
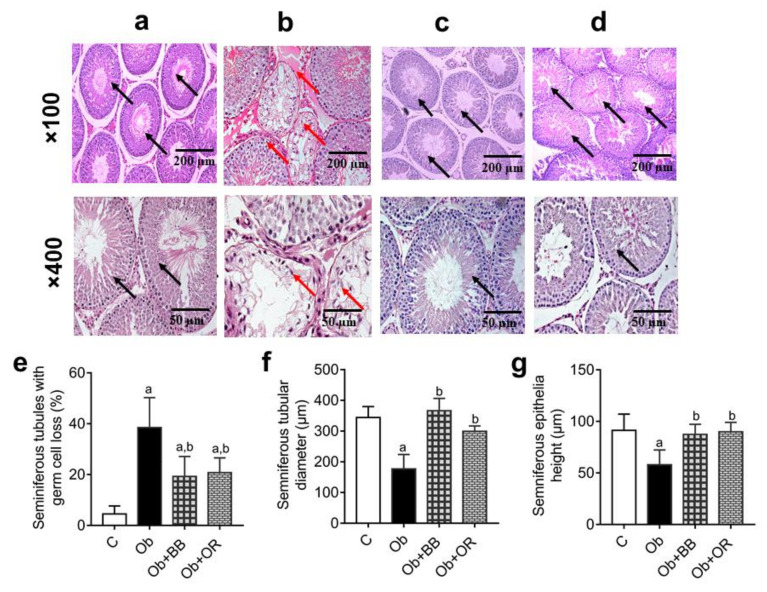
Representative photomicrographs of H&E staining of testes in (**a**) C, (**b**) Ob, (**c**), Ob + BB (**d**) Ob + OR groups. C: control; Ob: obese rats; Ob + BB: obese + bee bread (6 weeks after induction of obesity); and Ob + OR: obese + orlistat (6 weeks after induction of obesity). The seminiferous tubules in the Ob group were collapsed showing decreased amount of sperm and germ cells (red arrows) compared to C, Ob + BB and Ob + OR groups (**c**,**d**) which showed increased amount of sperm and germ cells as well as normal size seminiferous tubules (black arrows) (magnification of panel above: ×100, scale bar = 200 µm; magnification of panel below: ×400, scale bar = 50 µm). (**e**) Seminiferous tubule with germ cell loss, (**f**) seminiferous tubular diameter, and (**g**) seminiferous epithelial height are quantitative data, values are mean ± SD, *n* = 6. ^a^ *p* < 0.05 vs. C, ^b^ *p* < 0.05 vs. Ob (one-way ANOVA followed by Tukey post-hoc test).

The histological architecture of the testes revealed decreases in epithelial height and the diameter of seminiferous tubules, as well as the concentration of the sperm (red arrows) in the Ob group relative to the C group (*p* < 0.05) (black arrows) (Figure 1a–g). The administration of bee bread revealed marked improvements for these parameters in the Ob + BB group which were comparable to the orlistat-treated group.

Further, the Ob group revealed a significant increase in the percentage of seminiferous tubules with germ cell loss relative to the C group. Nevertheless, there was a significant decrease in the percentage of seminiferous tubules with germ cell loss in the Ob + BB group relative to the Ob group (*p* < 0.05), which was comparable to the Ob + OR group (Figure 1e).

### 3.6. Effects of Bee Bread on Testicular Oxidative Stress of Obese Rats

The activities of SOD, CAT, GST, GPx and GR, as well as the TAC level, were significantly lower in the Ob group relative to the C group (*p* < 0.05). However, the administration of bee bread showed that these activities and the level in the Ob + BB and Ob + OR groups were increased significantly in the Ob + BB group compared to the Ob group. In addition, the iNOS activity and MDA level were increased significantly in the Ob group relative to the C group. These were decreased significantly in the Ob + BB and Ob + OR groups (Table 6).

### 3.7. Effects of Bee Bread on mRNA Expressions of Testicular Oxidative Stress Markers of Obese Rats

**Figure 2:**
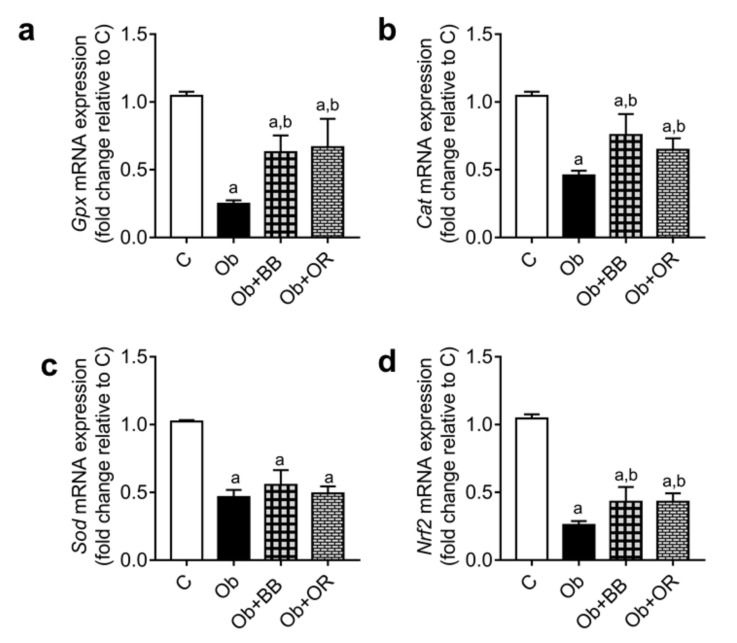
Effects of bee bread on the mRNA levels of oxidative stress markers; (**a**) *Gpx*, (**b**) *Cat*, (**c**) *Sod*, and (**d**) *Nrf2* in the testes of obese rats. *Gpx*: glutathione peroxidase, *Cat*: catalase, *Sod*: superoxide dismutase, *Nrf2*: nuclear factor erythroid 2-related factor 2, C: control, Ob: obese rats, Ob + BB: obese + bee bread (6 weeks after induction of obesity), and Ob + OR: Obese + orlistat (6 weeks after induction of obesity). Values are shown mean ± SD, *n* = 6. ^a^ *p* < 0.05 vs. C, ^b^ *p* < 0.05 vs. Ob (one-way ANOVA followed by Tukey’s post-hoc test).

*Gpx*, *Cat*, *Sod* and *Nrf2* mRNA transcript levels were decreased significantly in the Ob group relative to the C group. However, these genes, except for the *Sod* level, were significantly increased in the Ob + BB and Ob + OR groups relative to the Ob group (Figure 2).

### 3.8. Effects of Bee Bread on mRNA and Protein Levels of Testicular Inflammation-Related Markers of Obese Rats

**Figure 3:**
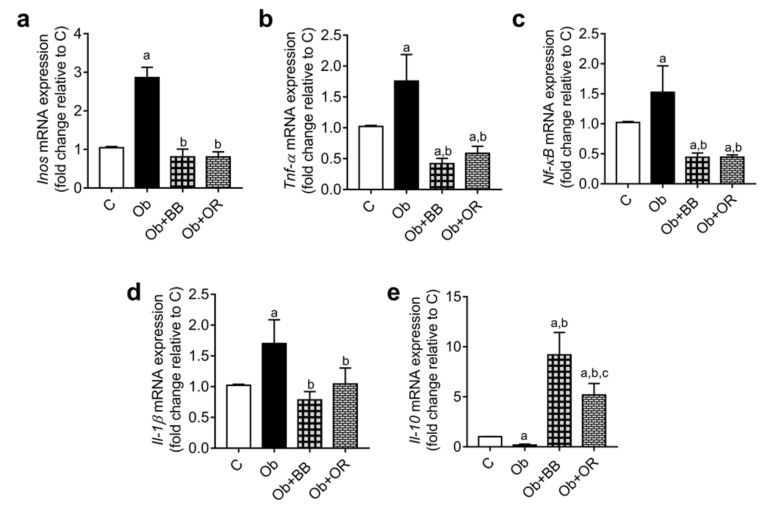
Effects of bee bread on the mRNA levels of inflammatory markers; (**a**) *Inos*, (**b**) *Tnf-α*, (**c**) *Nf-κB*, (**d**) *Il-1β* and (**e**) *Il-10* in the testes of obese rats. *Inos*: inducible nitric oxide synthase, *Il*: interleukin, *Nf-κB*: nuclear factor kappa B, *Tnf*-α: tumour necrotic factor-alpha, C: control, Ob: obese rats, Ob + BB: obese + bee bread (6 weeks after induction of obesity), and Ob + OR: Obese + orlistat (6 weeks after induction of obesity). Values are mean ± SD, *n* = 6. ^a^ *p* < 0.05 vs. C, ^b^ *p* < 0.05 vs. Ob, ^c^ *p* < 0.05 vs. Ob + BB (one-way ANOVA followed by Tukey’s post-hoc test).

*Tnf-α*, *Nf-κb(p65)*, *Il-1β* and *Inos* mRNA transcript levels increased significantly in the Ob group relative to the C group (*p* < 0.05). Meanwhile, the mRNA transcript levels of these parameters decreased significantly in the Ob + BB and Ob + OR groups. Additionally, the *Il-10* mRNA transcript level was decreased significantly in the Ob group relative to the C group (*p* < 0.05). Similarly, the *Il-10* mRNA transcript level significantly increased in the Ob + BB group relative to the Ob group (Figure 3a–e).

**Figure 4:**
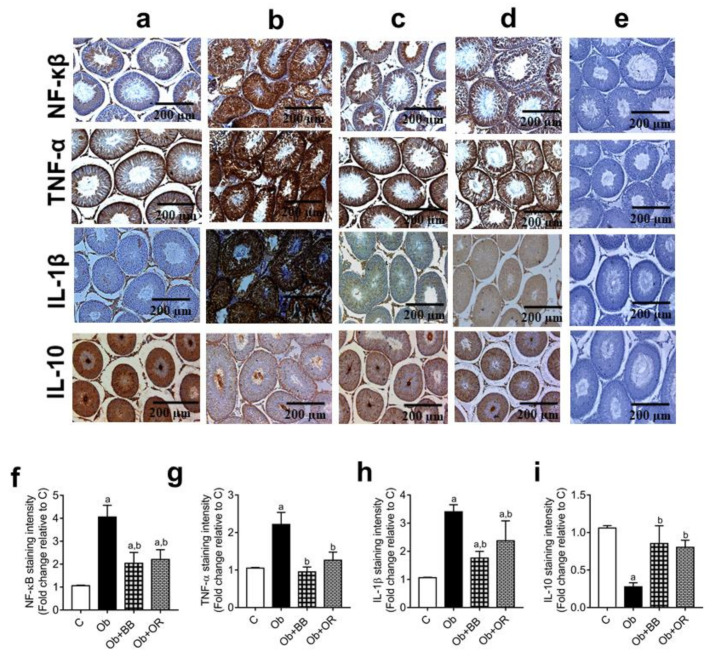
Effects of bee bread on immunoexpression of NF-κB, TNF-α, IL-1β and IL-10 proteins in the testes of obese rats. For each protein, C, Ob, Ob + BB and Ob + OR groups are represented by (**a**–**d**), respectively, while (**e**) represents the negative control (PBS). Staining using immunohistochemistry protocol showed seminiferous tubules with increased expressions of NF-κB, TNF-α and IL-1β, and decreased expression of IL-10 in Ob group, compared to C, Ob + BB and Ob + OR groups. Quantitative data are shown in (**f**) NF-κB, (**g**) TNF-α, (**h**) IL-1β and (**i**) IL-10. Values are mean ± SD, *n* = 6. ^a^ *p* < 0.05 vs. C, ^b^ *p* < 0.05 vs. Ob (one-way ANOVA followed by Tukey’s post-hoc test).

Furthermore, the immunoexpression of the pro-inflammatory proteins TNF-α, NF-kB(p65), and IL-1ß were significantly increased in the testis of the Ob group relative to the C group. Nevertheless, the above immunoexpressions decreased significantly in the Ob + BB group relative to the Ob group (*p* < 0.05), which was comparable to the Ob + OR group. On the other hand, the immunoexpression of the pro-inflammatory protein IL-10 was significantly decreased in the testis of the Ob group relative to the C group (*p* < 0.05). The administration of bee bread increased the immunoexpression of the IL-10 protein in the Ob + BB group relative to the Ob group, comparable to the orlistat-treated groups. Image J software was used to analyse the quantitative data of the area stained in the images of IHC expression (Figure 4a–i).

### 3.9. Effects of Bee Bread on mRNA and Protein Levels of Testicular Apoptosis-Related Markers and PCNA Expression of Obese Rats

**Figure 5:**
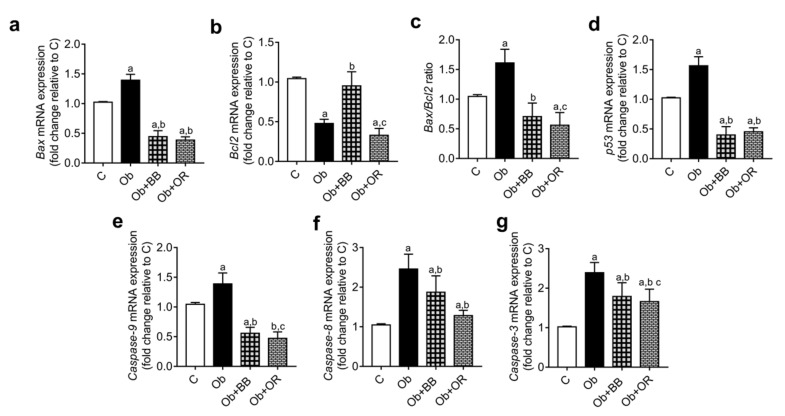
Effects of bee bread on the mRNA levels of apoptosis-related genes; (**a**) *Bax*, (**b**) *Bcl-2*, (**c**) Bax/Bcl2 ratio, (**d**) *p53*, (**e**) *caspase-9*, (**f**) *caspase-8* and (**g**) *caspase-3* in the testes of obese rats. p53: tumour suppressor, Bax: beta cell lymphoma 2 apoprotein X, Bcl2: beta cell lymphoma 2. Values are mean ± SD, *n* = 6. ^a^ *p* < 0.05 vs. C, ^b^ *p* < 0.05 vs. Ob, ^c^ *p* < 0.05 vs. Ob + BB (one-way ANOVA followed by Tukey’s post-hoc test).

*Caspases-3*, *8* and *9*, *Bax* and *p53* mRNA transcript levels were significantly increased in the Ob group relative to the C group. The mRNA levels of these parameters decreased significantly in the Ob + BB group relative to the Ob group, which was comparable with the Ob + OR group. Meanwhile, the Bcl2 mRNA level significantly decreased in the Ob group relative to the C group but increased significantly in the Ob + BB group which was comparable with the Ob + OR group. The Bax/Bcl2 ratio increased significantly in the Ob group relative to the C group. A significant decrease in this ratio was observed in the Ob + BB group relative to the Ob group (Figure 5a–g).

**Figure 6:**
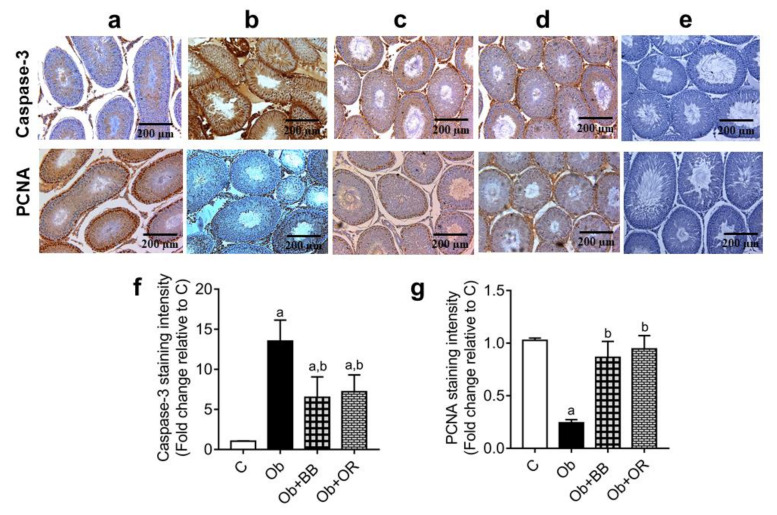
Effects of bee bread on immunoexpression of cleaved caspase-3 protein and proliferating cell nuclear antigen (PCNA) in the testes of obese rats. For each protein, C, Ob, Ob + BB, and Ob + OR groups are represented by (**a**–**d**), respectively, while (**e**) represents the negative control (PBS). Staining using immunohistochemistry protocol showed seminiferous tubules with increased cleaved caspase-3 and decreased PCNA levels in Ob group compared to C, Ob + BB, and Ob + OR groups (magnification = ×100, scale bar = 200 µm). Quantitative data are shown in (**f**) Caspase-3 staining intensity, and (**g**) PCNA-positive germ cells. Values are mean ± SD, *n* = 6. ^a^ *p* < 0.05 vs. C, ^b^ *p* < 0.05 vs. Ob (one-way ANOVA followed by Tukey’s post-hoc test).

The immunoexpression of PCNA positive germ cells was significantly decreased, and the immunoexpression of Caspase-3 was significantly increased in testis of the Ob group relative to the C group. There were significant increases in the immunoexpressions of PCNA positive germ cells and significant decreases in the immunoexpressions of Caspase-3 in the Ob + BB groups relative to the Ob group, which were comparable with orlistat-treated group (Figure 6a–g).

## 4. Discussion

Infertility in males is fast becoming the sole factor responsible for about 20% of fertility problems in couples all over the world [29]. This may be as a result of several causes including metabolic diseases such as obesity. Obesity is a debilitating metabolic disease that affects most of the major organs of the body. One of such organs severely affected is the testis [30]. We had previously reported that increased consumption of HFD causes excessive testicular oxidative stress, high levels of inflammation, and apoptosis in obese rat models fed with HFD for twelve weeks [12,31]. Consequently, the administration of bee bread for the same period prevented, to a large extent, the development of obesity, as well as considerable decreases in the above parameters, thereby establishing the ameliorative/preventive effect of bee bread [12]. This present study, however, concentrated on the therapeutic effects of bee bread in obese male rats fed with HFD. The rat model was designed to first induce obesity in rats for 6 weeks and, subsequently, they were administered with bee bread for another 6 weeks after the confirmation of obesity. The reason for the model was to determine if bee bread could reverse the debilitating effects of obesity in the testis, in which the oxidative stress, inflammation and apoptotic parameters were also assessed.

Our study revealed increases in final body weight, mean weight gain and daily weight gain in the obese rats which corroborates with our previous finding [12,31]. Upon treatment with bee bread, reduction in weight again was observed, indicating that bee bread might suppress the lipogenic effect of HFD which results in the accumulation of fats leading to increase in weight gain. Furthermore, this lipogenic effects may be due to presence of phenols and flavonoids present in bee bread [3].

Consequently, increases in the body mass index, Lee obesity index, and energy intake in the obese rats were observed in our present study. This may be as a result of increased fats deposits in the adipocytes and corroborates with the findings of our previous model of inducing obesity [12,31]. However, treatment with bee bread revealed decreases in the body mass and Lee obesity indexes in the bee bread-treated group, for the present model of inducing obesity, and is consistent with our previously reported study which could be traced to the presence of phenols and flavonoids in bee bread [3].

Further, HFD-induced obesity caused an increased accumulation of lipids in adipose tissue, as indicated by the data from the increased weight of the epididymal fat in our present study which might be related to triglyceride accumulation in adipocytes that seems to be a major source of oxidative stress in white adipose tissues and adipocytokine dysregulation, leading to metabolic syndrome. Our study also revealed altered levels of TG, TC, LDL-C and HDL-C in the obese rats. Treatment with bee bread improved these parameters in the bee bread-treated rats and corroborates with our previous findings [12,31], which may be due to high amounts of phenols and flavonoid present in bee bread [3].

Oxidative stress is one of the mechanisms involved in obesity where several organs in the body, including the male reproductive organs like the testis and epididymis, are badly affected. Oxidative stress is referred to as an increase in the rate of cellular damage caused by oxygen and oxygen-derived oxidants known as radical oxygen species (ROS). It is important to state that the main targets of ROS are the membrane lipids causing lipid peroxidation. Thus, during spermatogenesis and spermiogenesis, sperm cells are readily attacked by the ROS leading to lipid peroxidation. HFD has been implicated in oxidative stress in the testis as our studies [12,31] had earlier established that intake of HFD for 12 weeks induced testicular oxidative stress in male rats. Our present study also revealed increased testicular oxidative stress in the obese group which is in line with our previous study [12,31]. However, treatment with bee bread in the bee bread-treated group showed improvements in the oxidative status which may be due to the presence of active components like phenols and flavonoids, as well as some antioxidant properties found in bee bread [3].

Another very vital mechanism involved in the testis of obese males is inflammation. It is considered as an imbalance between the pro- and anti- inflammatory markers [49]. Previously, a group of cytokines were thought to be released during injuries and inflammation, especially by the immune system, but now they are known to actively regulate the immune system and inflammation [50]. In the male reproductive system, it is believed that cytokines, whose origin are from Sertoli cells, Leydig cells, seminal vesicles, the epididymis, prostate, testicular macrophages, and male accessory glands, accumulate in the seminal plasma, which ultimately affects steroidogenesis, spermatogenesis, and sperm function [51,52]. Intercellular and vascular adhesion molecules, as well as chemokines, are responsible for the secretion of necrotic fat cells which cause the proliferation and movement of monocytes and macrophages to adipose tissues [53,54].

Furthermore, pro-inflammatory markers are secreted by M1 macrophages, and activated by the NF-κB pathway, thereby increasing the release of pro-inflammatory markers, like interleukin-1β (IL-1β), interleukin-6 (IL-6), and TNF-α, as well as decreasing anti-inflammatory markers [55]. The above events lead to chronic inflammation. In addition, an increase in the size of adipocytes results in local hypoxia due to inadequate blood flow. The cell increases the production of hypoxia inducible factor-1α (HIF-1α) [56], heme-oxygenesase-1 (HO-1) [57], pyruvate dehydrogenase kinase-1 (PDK1) and vascular endothelial growth factor (VEGF) [58] to protect itself against this hypoxic state. Our present study showed increased testicular inflammation in the obese group, which is consistent with our study previously reported [12,31]. Treatment with bee bread reversed this trend in the bee bread-treated group which is consistent with our previous model of inducing obesity and may be attributed to the presence of phenols and flavonoids [3].

**Figure 7:**
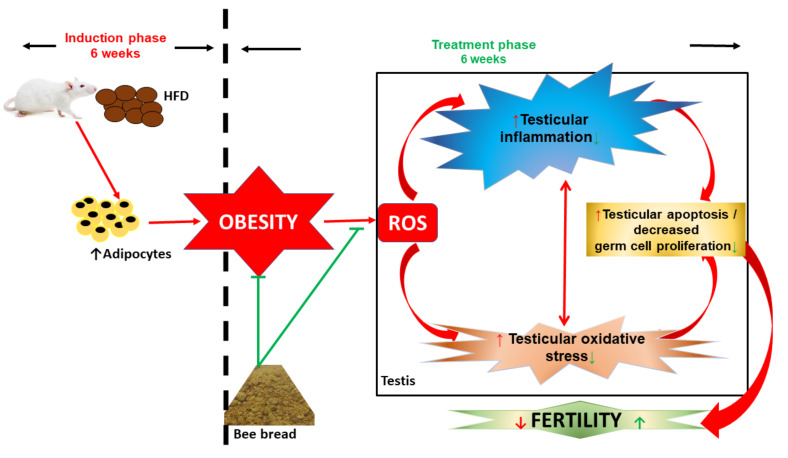
Schematic representation of therapeutic effects of bee bread on testicular-derived oxidative stress, inflammation, and apoptosis. HFD: high-fat diet, ROS reactive oxygen species.

Apoptosis is an organised cell death which helps in eliminating unwanted cells or damaged beyond repair [59]. The activation of Bcl-XL and Bax helps in maintaining the pro-apoptotic and anti-apoptotic markers [60,61]. Several studies have reported that one of the main causes of infertility in men is the imbalance between the pro- and anti-apoptotic factors [62,63]. In an obese state, the expression of Bcl-2 is suppressed, while that of Bax is significantly increased which leads to the caspase pathway activation [64]. Moreover, an increased level of lipids results in an increased level of stress which induces apoptosis in sperm cells with the increased expression of the binding immunoglobulin protein [65]. Our present study revealed increased testicular apoptosis in the obese group, which is consistent with our previous study [12,31]. However, treatment with bee bread in the bee bread-treated group caused reversal in apoptosis, suggesting an anti-apototic property of bee bread which may be due to the presence of active components like phenols and flavonoids in bee bread [3]. Therefore, the therapeutic effects of bee bread on testicular-derived oxidative stress, inflammation and apoptosis are summarised in Figure 7.

## 5. Conclusions

Conclusively, our study has revealed that the consumption of HFD leads to obese conditions and poses a great danger to the body, including the male reproductive system. Secondly, it is now obvious that bee bread has therapeutic effects against oxidative stress, inflammation, and apoptosis generated by HFD in the testis in obese rats. This might partly be due to the presence of its constituents, which is suggested for further study to evaluate the exact molecular mechanism of action. Furthermore, a clinical trial is recommended to ascertain the use of bee bread for the possible treatment of infertility/sub-infertility in men with fertility problems.

## Figures and Tables

**Table 1:** Sequence for Primers in tabular form.

		Primer Sequence (5′–3′)		
Gene	Accession Number	Forward	Reverse	Amplicon Size (bp)
CAT	NM_012520.2	ACAACTCCCAGAAGCCTAAGAATG	GCTTTTCCCTTGGCAGCTATG	76
Caspase-8	NM_022277.1	GTTCTCTCAGTTGCCTTTCTCC	GGCCAGTCCGCCAAAGTTTA	90
IL-10	NM_012854.2	TTGAACCACCCGGCATCTAC	CCAAGGAGTTGCTCCCGTTA	91
Inos	XM_006246949.3	CAGCCCTCAGAGTACAACGAT	CAGCAGGCACACGCAATGAT	91
SOD	X05634.1	CGAGCATGGGTTCCATGTC	CTGGACCGCCATGTTTCTTAG	101
IL-1b	NM_031512.2	GACTTCACCATGGAACCCGT	GGAGACTGCCCATTCTCGAC	104
p53	NG_005120.4	CTACTAAGGTCGTGAGACGCTGCC	TCAGCATACAGGTTTCCTTCCACC	106
Nrf2	NM_031789.1	CAGGTTGCCCACATTCCCAA	ATATCCAGGGCAAGCGACTCAT	110
Bax	U49729.1	CGCGTGGTTGCCCTCTTCTACTTT	CAAGCAGCCGCTCACGGAGGA	129
Bcl-2	NM_016993.1	ATCGCTCTGTGGATGACTGAGTAC	AGAGACAGCCAGGAGAAATCAAAC	134
GPx	NM_030826.4	GGAGAATGGCAAGAATGAAGA	CCGCAGGAAGGTAAAGAG	139
TNF-a	NM_012675.3	ACTGAACTTCGGGGTGATCG	GCTTGGTGGTTTGCTACGAC	153
NF-kB(p65)	NM_199267.2	CGCGGGGACTATGACTTGAA	AGTTCCGGTTTACTCGGCAG	163
Caspase-9	NM_031632	CTGAGCCAGATGCTGTCCCATA	CCAAGGTCTCGATGTACCAGGAA	168
GAPDH	NM_017008	TCACCACCATGGAGAAGGC	GCTAAGCAGTTGGTGGTGCA	169
Caspase-3	NM_012922	AAGATACCAGTGGAGGCCGACTTC	GGGAGAAGGACTCAAATTCCGTGG	199

CAT: catalase; iNOS; inducible nitric oxide synthase; SOD: superoxide dismutase; IL: interleukin; p53: tumour suppressor; Nrf2: nuclear factor erythroid 2-related factor 2; Bcl2: B-cell lymphoma 2; Bax: Bcl-2-associated X protein; GPx: glutathione peroxidase; TNF: tumour necrosis factor; NF-kB: nuclear factor kappa B; GAPDH: glyceraldehyde-3-phosphate dehydrogenase.

**Table 2:** Body weight and related parameters in all groups.

Parameter	C	Ob	Ob + BB	Ob + OR
Initial body weight	242.20 ± 34.47	256.10 ± 47.24	271.90 ± 14.14	253.50 ± 36.20
Final body weight (g)	386.80 ± 40.11	444.90 ± 38.62 ^a^	411.90 ± 36.22	405.30 ± 41.25
Mean weight gain (g)	144.70 ± 53.51	188.90 ± 44.80 ^a^	140.10 ± 32.46 ^b^	151.80 ± 19.18
Mean daily weight gain (g/day)	1.72 ± 0.64	2.25 ± 0.53	1.67 ± 0.39	1.81 ± 0.23

C: normal control, Ob: obese rats, Ob + BB: obese rat fed with high-fat diet plus bee bread (6 weeks), Ob + OR: obese rat fed with high-fat diet plus orlistat (6 weeks). Data are shown as mean ± SD, *n* = 8/group. ^a^ *p* < 0.05 vs. C, ^b^ *p* < 0.05 vs. Ob. (One-way ANOVA followed by Tukey post-hoc test).

**Table 3:** Energy intake, feed consumption, Lee obesity index, and Body mass index in all groups.

Parameter	C	Ob	Ob + BB	Ob + OR
Lee obesity index	305.90 ± 5.88	323.10 ± 8.91 ^a^	304.60 ± 8.12 ^b^	309.10 ± 6.01 ^b^
BMI (gcm^−1^)	0.71 ± 0.04	0.83 ± 0.06 ^a^	0.72 ± 0.04 ^b^	0.73 ± 0.05 ^b^
Total feed consumption (g)	1822.00 ± 125.80	1647.00 ± 266.20	1560.00 ± 171.40	1628.00 ± 232.80
Mean food consumption (g)	21.69 ± 1.49	19.61 ± 3.17	18.57 ± 2.04	19.38 ± 2.77
Energy intake (kcal/day)	69.15 ± 4.77	101.30 ± 16.37 ^a^	95.91 ± 10.53 ^a^	100.10 ± 14.31 ^a^

BMI: body mass index, C: normal control, Ob: obese rats, Ob + BB: obese rat fed with high-fat diet plus bee bread (6 weeks), Ob + OR: obese rat fed with high-fat diet plus orlistat (6 weeks). Data are shown as mean ± SD, *n* = 8/group. ^a^ *p* < 0.05 vs. C, ^b^ *p* < 0.05 vs. Ob (One-way ANOVA followed by Tukey post-hoc test).

**Table 4:** Reproductive organs and epididymal fat weights in all groups.

Parameter		C	Ob	Ob + BB	Ob + OR
Testis ^≠^	AW (g)	3.75 ± 0.26	3.34 ± 0.58 ^a^	3.52 ± 0.29	3.57 ± 0.57
	RW (%)	1.00 ± 0.09	0.79 ± 0.19 ^a^	0.82 ± 0.11	0.76 ± 0.17 ^a^
Epididymis ^≠^	AW (g)	1.52 ± 0.28	1.32 ± 0.29	1.51 ± 0.22	1.44 ± 1.17
	RW (%)	0.40 ± 0.07	0.31 ± 0.09	0.35 ± 0.05	0.31 ± 0.06 ^a^
Prostate	AW (g)	1.22 ± 0.43	1.52 ± 0.98	1.53 ± 0.61	1.68 ± 0.40
	RW (%)	0.31 ± 0.11	0.34 ± 0.20	0.34 ± 0.12	0.35 ± 0.09
Seminal Vessicle	AW(g)	1.76 ± 0.30	1.97 ± 0.57	2.11 ± 0.41	2.00 ± 0.52
	RW (%)	0.47 ± 0.08	0.45 ± 0.11	0.48 ± 0.06	0.42 ± 0.12
Penis	AW (g)	0.27 ± 0.04	0.24 ± 0.03 ^a^	0.27 ± 0.05	0.30 ± 0.03 ^b^
	RW (%)	0.07 ± 0.01	0.06 ± 0.01	0.06 ± 0.01	0.06 ± 0.01
Epididymal Fat	AW (g)	3.77 ± 1.33	10.38 ± 4.27 ^a^	7.87 ± 5.89 ^b^	9.54 ± 3.92 ^a^
	RW (%)	0.99 ± 0.28	2.33 ± 0.74 ^a^	1.74 ± 1.08	1.92 ± 0.54

^≠^ Data are the combined weights of the paired organs. AW: absolute weight; RW: relative weight; C: normal control; Ob: obese rats; Ob + BB: obese rat fed with high-fat diet plus bee bread (6 weeks); Ob + OR: obese rat fed with high-fat diet plus orlistat (6 weeks). Data are shown as mean ± SD, *n* = 8/group. ^a^ *p* < 0.05 vs. C, ^b^ *p* < 0.05 vs. Ob (One-way ANOVA followed by Tukey post-hoc test).

**Table 5:** Lipid profile parameters in all groups.

Parameter	C	Ob	Ob + BB	Ob + OR
TC (mmol/L)	1.61 ± 0.18	2.36 ± 0.76 ^a^	1.89 ± 0.20	2.11 ± 0.17
TG (mmol/L)	0.50 ± 0.07	0.93 ± 0.09 ^a^	0.71 ± 0.15	0.93 ± 0.28 ^a^
HDL-C (mmol/L)	0.42 ± 0.10	0.22 ± 0.15 ^a^	0.40 ± 0.04 ^b^	0.37 ± 0.09
LDL-C (mmol/L)	0.63 ± 0.05	1.56 ± 0.15 ^a^	1.25 ± 0.14 ^a,b^	1.35 ± 0.21 ^a^

TC: total cholesterol, TG: triglyceride, HDL: high-density lipoproteins, LDL: low-density lipoproteins, C: normal control, Ob: obese rats, Ob + BB: obese rat fed with high-fat diet plus bee bread (6 weeks), Ob + OR: obese rat fed with high-fat diet plus orlistat (6 weeks). Data are shown as mean ± SD, *n* = 8/group. ^a^ *p* < 0.05 vs. C, ^b^ *p* < 0.05 vs. Ob (One-way ANOVA followed by Tukey post-hoc test).

**Table 6:** Oxidant–antioxidant Parameters in the testis in all groups.

Parameters	C	Ob	Ob + BB	Ob + OR
CAT activity (unit/mg protein)	32.88 ± 3.92	14.86 ± 3.29 ^a^	25.80 ± 3.11 ^a,b^	23.12 ± 1.63 ^a,b^
GPx activity (unit/mg protein)	43.60 ± 2.19	20.13 ± 1.88 ^a^	41.30 ± 3.14 ^b^	37.79 ± 1.80 ^a,b,c^
GR activity (unit/mg protein)	43.92 ± 2.62	16.99 ± 1.79 ^a^	46.71 ± 6.53 ^b^	42.26 ± 3.77 ^b^
GSH (mmol GSH Eq/mg protein)	10.73 ± 1.60	19.76 ± 5.41 ^a^	13.94 ± 2.38 ^b^	13.83 ± 5.00 ^b^
GST activity (unit/mg protein)	257.20 ± 26.93	151.40 ± 17.32 ^a^	272.2 ± 24.37 ^b^	250.70 ± 32.41 ^b^
iNOS activity (ng/mL protein)	1.80 ± 0.63	7.31 ± 1.06 ^a^	3.01 ± 0.79 ^a,b^	1.80 ± 0.51 ^b^
MDA (nmol/mg protein)	1.27 ± 0.12	12.01 ± 0.76 ^a^	1.70 ± 0.28 ^b^	1.38 ± 0.33 ^b^
SOD activity (unit/mg protein)	2.78 ± 0.12	0.29 ± 0.05 ^a^	2.31 ± 0.08 ^b^	2.31 ± 0.09 ^b^
TAC (nmol uric acid Eq/mg protein)	137.90 ± 4.64	63.64 ± 6.01 ^a^	131.30 ± 10.85 ^b^	128.70 ± 6.74 ^b^

TAC: total antioxidant capacity, SOD: superoxide dismutase, MDA: malondialdehyde, GPx: glutathione peroxidase, GST: glutathione-S-transferase, GSH: total glutathione, CAT: catalase, GR: glutathione reductase, C: normal control, Ob: obese rats, Ob + BB: obese rat fed with high-fat diet plus bee bread (6 weeks), Ob + OR: obese rat fed with high-fat diet plus orlistat (6 weeks). Data are presented as mean ± SD, *n* = 8/group. ^a^ *p* < 0.05 vs. C, ^b^ *p* < 0.05 vs. Ob, ^c^ *p* < 0.05 vs. Ob + BB (One-way ANOVA followed by Tukey post-hoc test).

## Data Availability

All data is contained within the present article.
